# Mitochondrial Function in Hereditary Spastic Paraplegia: Deficits in *SPG7* but Not *SPAST* Patient-Derived Stem Cells

**DOI:** 10.3389/fnins.2020.00820

**Published:** 2020-08-20

**Authors:** Gautam Wali, Kishore Raj Kumar, Erandhi Liyanage, Ryan L. Davis, Alan Mackay-Sim, Carolyn M. Sue

**Affiliations:** ^1^Department of Neurogenetics, Royal North Shore Hospital, Kolling Institute of Medical Research, The University of Sydney, Sydney, NSW, Australia; ^2^Faculty of Medicine and Health, Sydney Medical School, The University of Sydney, Sydney, NSW, Australia; ^3^Molecular Medicine Laboratory, Department of Neurology, Concord Hospital, Sydney, NSW, Australia; ^4^Kinghorn Centre for Clinical Genomics, Garvan Institute of Medical Research, Sydney, NSW, Australia; ^5^Department of Neurology, Royal North Shore Hospital, Northern Sydney Local Health District, Sydney, NSW, Australia; ^6^Griffith Institute for Drug Discovery, Griffith University, Brisbane, QLD, Australia

**Keywords:** hereditary spastic paraplegia, *SPG7*, *SPAST*, spastin, paraplegin, mitochondria, oxidative phosphorylation

## Abstract

Mutations in *SPG7* and *SPAST* are common causes of hereditary spastic paraplegia (HSP). While some *SPG7* mutations cause paraplegin deficiency, other *SPG7* mutations cause increased paraplegin expression. Mitochondrial function has been studied in models that are paraplegin-deficient (human, mouse, and *Drosophila* models with large exonic deletions, null mutations, or knockout models) but not in models of mutations that express paraplegin. Here, we evaluated mitochondrial function in olfactory neurosphere-derived cells, derived from patients with a variety of *SPG7* mutations that express paraplegin and compared them to cells derived from healthy controls and HSP patients with *SPAST* mutations, as a disease control. We quantified paraplegin expression and an extensive range of mitochondrial morphology measures (fragmentation, interconnectivity, and mass), mitochondrial function measures (membrane potential, oxidative phosphorylation, and oxidative stress), and cell proliferation. Compared to control cells, *SPG7* patient cells had increased paraplegin expression, fragmented mitochondria with low interconnectivity, reduced mitochondrial mass, decreased mitochondrial membrane potential, reduced oxidative phosphorylation, reduced ATP content, increased mitochondrial oxidative stress, and reduced cellular proliferation. Mitochondrial dysfunction was specific to *SPG7* patient cells and not present in *SPAST* patient cells, which displayed mitochondrial functions similar to control cells. The mitochondrial dysfunction observed here in *SPG7* patient cells that express paraplegin was similar to the dysfunction reported in cell models without paraplegin expression. The p.A510V mutation was common to all patients and was the likely species associated with increased expression, albeit seemingly non-functional. The lack of a mitochondrial phenotype in *SPAST* patient cells indicates genotype-specific mechanisms of disease in these HSP patients.

## Introduction

Hereditary spastic paraplegia (HSP) is an inherited, progressive neurodegenerative condition causing spasticity and weakness in the lower limbs as a consequence of corticospinal tract degeneration (Hedera et al., 2005; Salinas et al., 2008; Kumar et al., 2015). Mutations in *SPG7* and *SPAST* are the most common causes of HSP. *SPG7* encodes for paraplegin, a mitochondrial matrix protease embedded in the inner mitochondrial membrane (Casari et al., 1998). Paraplegin is involved in multiple mitochondrial processes including mitochondrial protein quality surveillance, ribosome assembly, and mitochondrial biogenesis (Quirós et al., 2015). *SPG7* mutations can cause loss of paraplegin (Atorino et al., 2003) or increased paraplegin expression (Thal et al., 2015; Pfeffer et al., 2014). The effects of *SPG7* mutations leading to a loss of paraplegin expression on mitochondrial function have been characterized. For example, fibroblasts derived from a patient carrying a homozygous 9.5-kb deletion within the *SPG7* gene that caused paraplegin deficiency exhibited impaired respiratory chain complex I activity. When the cells were exposed to hydrogen peroxide, to stress mitochondrial function, they were found to have lower ATP synthesis rate and reduced cell viability (Atorino et al., 2003). Consistent with this, a *SPG7*-null mutant *Drosophila* model displayed reduced activity of respiratory chain complexes I and II, severely swollen and aberrantly shaped mitochondria in the synaptic terminals of photoreceptors, and neuronal and muscular degeneration (Pareek et al., 2018).

There is limited information on mitochondrial function in patient cells and tissues with *SPG7* mutations that do not cause a loss of paraplegin. Post-mortem analysis of a patient with homozygous p.Ala510Val mutations showed an increased expression of paraplegin in neurites of the cerebellar and cerebral cortex (Thal et al., 2015), but mitochondria function was not evaluated. In *SPG7* patients carrying compound heterozygous mutations with increased paraplegin expression, the skeletal muscle biopsies showed increased mitochondrial biogenesis, multiple mitochondrial DNA deletions, and cytochrome *c* oxidase-deficient fibers (Pfeffer et al., 2014). Mitochondrial morphology evaluation of fibroblasts from these patients showed an increased mitochondrial mass and an increased number and length of mitochondrial networks; however, mitochondrial respiratory chain function was not evaluated. Inhibition of the respiratory chain function can lead to excess generation of reactive oxygen species, subsequently leading to cell death mediated by oxidative stress (Keane et al., 2011). This link has been shown for many neurodegenerative diseases (Moreira et al., 2010; Keane et al., 2011). Here, we characterize mitochondria morphology, respiratory chain function, and oxidative stress in paraplegin expressing *SPG7* patient cells.

*SPAST* encodes for spastin, a microtubule-severing protein (Errico et al., 2002; Orlacchio et al., 2004). *SPAST* patient cells have impaired organelle transport, which includes mitochondria (Abrahamsen et al., 2013; Wali et al., 2020). However, it remains to be determined whether mitochondrial respiratory chain function is affected in *SPAST* patient cells. A study investigating muscle biopsies from *SPAST* patients did not show any aberrant activity of complexes I, II, and IV or citrate synthase (McDermott et al., 2003). Histochemical reactions, cytochrome *c* oxidase, and succinate dehydrogenase were also normal (McDermott et al., 2003). However, a recent study in a single *SPAST* patient fibroblast cell line showed that the patient cells had less tubular and more fragmented mitochondria, reduced mitochondrial membrane potential, and altered mitochondrial distribution (Dong et al., 2018).

In the present study, we quantified paraplegin expression and a range of mitochondrial morphology measures (fragmentation, interconnectivity, and mass), mitochondrial functions (membrane potential, oxidative phosphorylation, and oxidative stress), and cell proliferation. These measures were performed on olfactory neurosphere-derived stem cells (ONS cells) derived from HSP patients with *SPG7* mutations, from healthy controls, and from HSP patients with *SPAST* mutations.

## Materials and Methods

### Participants

*SPG7* and *SPAST* HSP patients involved in this study were examined by neurologists CMS and KRK. Details of the patient clinical characteristics and causative gene mutations are listed in Table 1. Control and patient samples used were matched for age and gender. Mean age range of *SPG7* patients was 60 years, and those of *SPAST* patients and healthy controls were 51 and 60 years, respectively. Olfactory neurosphere-derived cells (ONS cells) were prepared from olfactory mucosa biopsies of participants (Féron et al., 1998, 2013) with their informed and written consent.

**TABLE 1:** Clinical features and mutation details of HSP patients with *SPG7* and *SPAST* mutations.

Participant ID	L-6709*	L-7632*	N22090010	610080001^#^	610080002^#^	610080006^#^
Gene	*SPG7*	*SPG7*	*SPG7*	*SPAST*	*SPAST*	*SPAST*
Mutation(s)	c.1529C > T (het), c.1454_1462del (het)	c.1529C > T (het), c.1727C > G (het)	c.1529C > T (het), c.1449 + 1G > A (het) (novel)	c.1392A > T	c.583C > G	c.1096G > A
Patient ID	Patient 1	Patient 2	Patient 3	Patient 4	Patient 5	Patient 6
Ethnic background	European	European	European	European	European	European
Phenotype	Complex	Complex	Complex	Complex	Pure	Pure
Family History	Sporadic	AR	AR	Sporadic	AD	AD
UL amyotrophy	No	No	No	No	No	No
UL spasticity	No	No	No	Moderate	No	No
UL weakness	No	No	No	Yes	No	No
UL reflexes	+++	++	++	+++	+++	+++
LL amyotrophy	No	No	No	No	No	No
LL spasticity	Moderate	Moderate	Severe	Severe	Mild	Moderate
LL weakness	Mild	No	Moderate	Severe	Mild	Moderate
LL reflexes	++++	++++	+++	++++	+++	+++
Babinski	Yes	Yes	Yes	Yes	No	Yes
Sensory disturbance	Temp/Noci	No	No	Vibr/Temp/Noci	No	Vibr
Urinary disturbance	No	Yes, urgency	Yes, urinary incontinence	Yes, suprapubic catheter	No	Yes
Ophthalmoplegia	Yes, bilateral external ophthalmoplegia with limitation of upgaze	No	No	No	No	No
Ptosis	Yes, mild bilateral ptosis	No	No	No	No	No
Dysarthria	No	No	Yes	Yes	No	No
Cerebellar ataxia	No	Yes	Yes	No	No	No
Walking aids	Walking stick	No	Wheelchair	Wheelchair	No	Walking stick
Brain MRI	Normal	Normal	Cerebellar atrophy	Normal	N/A	N/A
Spinal MRI	Normal	Mild cervical spinal stenosis, with no improvement following cervical decompression.	N/A	Cervical spondylosis	N/A	N/A
NCS/EMG	Normal	Normal	N/A	Normal	N/A	N/A
MEP	Normal for the UL, absent for the lower limb	Normal for the UL, prolonged CMCT for the LL (27.65, *N* < 13)	Normal for the UL, prolonged CMCT for the LL (17.35, *N* < 13)	N/A	N/A	N/A
Muscle biopsy features of mitochondrial cytopathy	Not performed	Yes, increased COX-negative/SDH-positive fibers for the patient’s age	Not performed	Not performed	Not performed	Not performed

UL, upper limb; LL, lower limb; M, male; F, female; Vibr/Prop/Temp/Noci impairment of vibration sense/proprioception/temperature sensation/nociception, CMCT ms central motor conduction time in milliseconds, N/A not available. Reflex grading: 0 absent, + reduced, ++ normal, +++ brisk without clonus, ++++ brisk with clonus. *Previously reported by Kumar et al. (2013). ^#^Previously reported by Abrahamsen et al. (2013) and Vandebona et al. (2012).

### Targeted Next-Generation Sequencing

All patients except patient 3 have been previously reported (refer to Table 1). Patient 3 was identified using targeted next-generation sequencing, as described elsewhere (Kumar et al., 2013). In brief, patients with an apparent autosomal recessive or sporadic mode of inheritance were screened for nine autosomal recessive HSP-associated genes using a PCR-based library preparation and Roche Junior 454 next-generation sequencing, after exclusion of *SPAST* mutations. Amplicons that did not achieve 30 reads were resequenced with a conventional Sanger sequencing method. All pathogenic or potentially pathogenic variants were confirmed by Sanger sequencing on an ABI Prism 3130xl Genetic Analyzer platform (Applied Biosystems).

### Cell Culture

ONS cells were derived from olfactory mucosa biopsies using our published techniques (Matigian et al., 2010; Féron et al., 2013). Cells were cultured in Dulbecco’s Modified Eagle Medium (DMEM)/F12 (Gibco) with 10% fetal bovine serum at 37°C and 5% CO_2_. Please refer to our previous publication (Féron et al., 2013) for a detailed protocol of generating olfactory neurosphere-derived cells from olfactory mucosal biopsies.

### Paraplegin Expression

#### Protein Isolation

Cells cultured in T-25 flasks were washed three times with Dulbecco’s phosphate-buffered saline (Gibco 14190250) to completely remove culture media. Cells were lysed with cell lysis buffer (Sigma, C3228) supplemented with a protease inhibitor (Thermo Fisher Scientific, 78429). Cells were harvested by scraping and maintained on ice over a rocker for 30 min. Then, the samples were centrifuged at 10,000 *g* for 10 min at 4°C. The protein samples were then collected and quantified using the BCA pierce TM BCA Protein Assay kit (Thermo Fisher Scientific, 23225).

#### Western Blot

Total cellular protein (20 μg) was resolved on a 12% Bis-Tris polyacrylamide gel and transferred onto a nitrocellulose membrane. The membrane blot was incubated overnight at 4°C with the primary mouse anti-human *SPG7* monoclonal antibody (dilution 1/10,000; OriGene). Following this, the membrane was incubated with goat anti-mouse secondary antibody coupled to horseradish peroxidase (dilution 1/4,000, Sigma-Aldrich). Bands were visualized using Pierce SuperSignal West Femto Chemiluminescent Substrate on a Bio-Rad ChemiDoc XL. Paraplegin expression was normalized to GAPDH (dilution 1/10,000; Abcam) expression levels using ImageJ Version 1.52u software (Schneider et al., 2012).

#### Western Blot Positive Control

Human HEL293 cells overexpressing paraplegin were used to validate the antibody (OriGene, LC401085). The paraplegin band size was 88 kDa for both the control samples tested and the positive control, showing antibody specificity (Supplementary Figure S1).

#### Flow Cytometry

Cells were fixed and permeabilized with the BD Cytofix/Cytoperm reagent for 20 min at room temperature. Cells were washed with BD Perm/Wash buffer and then incubated with mouse anti-human *SPG7* monoclonal antibody (dilution 1/10,000; OriGene) or the IgG1 isotype control for 1 h. Cells were washed and incubated with Alexa Fluor secondary antibody for 30 min. Cells were washed, and the relative fluorescence intensity was measured for 10,000 events per sample on a BD LSRFortessa, and the data were analyzed using BD FACS Diva software. Mean fluorescence intensity was calculated as the fluorescence intensity of the paraplegin antibody to the intensity of the isotype control antibody.

### Mitochondrial Morphology

Cells were treated with 100 nM MitoTracker green for 30 min at 37°C to label active mitochondria (Park et al., 2014). Images of mitochondria-labeled cells were captured using a 63× objective on a confocal microscope. Using ImageJ image analysis software, raw images were binarized, and mitochondrial morphological characteristics of the aspect ratio (AR), indicating mitochondrial length, form factor (FF), and mitochondrial branching, were calculated as described before (Mortiboys et al., 2008). AR is the ratio of the major to minor axis of the mitochondria, and FF is calculated as (perimeter of mitochondria^2^)/(4π ^∗^ area of mitochondria) (Mortiboys et al., 2008). Thirty cells were imaged and analyzed per cell line per technical replicate experiment.

### Mitochondrial Mass and Mitochondrial Membrane Potential

Mitochondrial mass and membrane potential were assessed by labeling mitochondria with 100 nM MitoTracker green for 30 min (as above) and 50 nM non-quenching TMRM for 30 min at 37°C, respectively. The cells were washed twice with PBS before measuring fluorescence using a BD LSRFortessa^™^. Fluorescence was measured for 10,000 events per sample, and the data were analyzed using the BD FACS Diva^™^ software (Gautier et al., 2012). Mitochondrial membrane potential was normalized to MitoTracker green fluorescence, a relative estimate of the mitochondrial mass per cell.

### MitoSOX and CM-H_2_DCFDA

ONS cells (10,000) were plated in each well of a black-walled 96-well plate and cultured for 24 h before being assessed. The cells were stained with 5 μM MitoSOX Red for 15 min (as an indication of mitochondrial oxidative stress) (Teves et al., 2018) or 5 μM CM-H_2_DCFDA for 15 min (a measure of general cellular oxidative stress) (Wali et al., 2016) at 37°C. The cells were washed twice with PBS before fluorescence was measured using a VICTOR Nivo Multimode Microplate Reader. MitoSOX and CM-H_2_DCFDA fluorescence levels were normalized to mitochondrial mass, using dual MitoTracker green staining.

### ATPlite Assay Measuring ATP Content

ONS cells (10,000 per well) were plated in a white-walled 96-well plate for 24 h before being assessed. The assay was performed as per the manufacturer’s instructions and as described before (Wali et al., 2016). Luminescence was measured using the VICTOR Nivo Multimode Microplate Reader. The ATPlite luminescence values were normalized to mitochondrial mass, using dual MitoTracker green staining.

### Oxygen Consumption Rate

A Seahorse XF24-3 Bioanalyzer (Agilent Technologies) was used to measure mitochondrial oxygen consumption as described previously (Koentjoro et al., 2017). On day 1, 70,000 ONS cells per well were plated in a 24-well XF24 V7 cell culture microplate and cultured in DMEM/F12 (Gibco) with 10% fetal bovine serum at 37°C and 5% CO_2_ for 24 h. On day 2, the DMEM/F12 media was replaced with Seahorse XF Base media supplemented with 2 mM L-glutamine, and the pH was adjusted to 7.35 ± 0.05 (basal media). Cells were incubated in the basal media for 1 h at 37°C in a non-CO_2_ incubator. The plate was then assembled with a hydrated sensor cartridge and placed in the Seahorse flux analyzer. The oxygen consumption rate (OCR) was measured at baseline and after subsequent stepwise injection of the mitochondrial respiratory chain inhibitors, oligomycin (1 μM), carbonyl cyanide-4-(trifluoromethoxy)phenylhydrazone (FCCP) (1 μM) and rotenone/antimycin A (0.5 μM). The OCR readings were normalized to cell number measured by CyQUANT assay in a sister plate. Multiple parameters of mitochondrial respiration were calculated based on the response of the cells to the respiratory chain inhibitors: (a) basal respiration, basal OCR - non-mitochondrial respiration rate; (b) ATP production, difference in OCR before and after oligomycin treatment; (c) maximal respiration, OCR measurement after FCCP treatment - OCR measurement after oligomycin treatment; and (d) spare respiratory capacity, maximal respiration - basal respiration.

### Cell Proliferation

ONS cells (5,000) were seeded into each well of a 96-well plate. On day 0 (4 h post seeding), day 1 (24 h post seeding), and day 2 (48 h post seeding), the relative cell numbers were assessed using the CyQUANT^®^ NF Cell Proliferation Assay Kit, following the manufacturer’s instructions.

### Statistical Analysis

Three technical replicates were performed for all experiments: three control cell lines, three *SPG7* patient lines, and three *SPAST* patient cell lines. One-way analysis of variance with Tukey’s multiple comparisons test was performed to compare control, *SPG7*, and *SPAST* sample results. Statistical analysis and graphing were performed with GraphPad (Prism 8).

## Results

### Clinical Phenotype

All patients with mutations in *SPG7* had complex HSP phenotypes (clinical features detailed in Table 1). Complicating features in *SPG7* patients included ophthalmoplegia (patient 1), ptosis (patient 1), ataxia (patients 2 and 3), and mitochondrial cytopathy on muscle biopsy (patient 1). In contrast, no *SPAST* patients (patients 4, 5, and 6) had typical mitochondrial disorder-related clinical signs (such as ocular myopathy, present in a range of mitochondrial myopathy syndromes; Pfeffer and Chinnery, 2013), and only one of three (patient 4) had additional features of dysarthria and upper limb weakness indicative of complicated HSP.

### Genetic Studies

**FIGURE 1:**
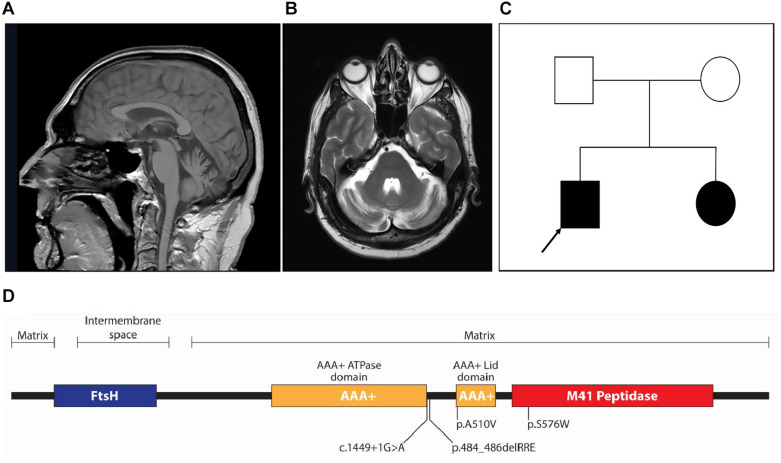
**(A)** Sagittal T1-weighted fast field echo and **(B)** transverse T2-weighted MRI images showing mild cerebellar atrophy of the patient (patient 3) carrying novel *SPG7* variant. **(C)** Pedigree of patient 3 (arrow); both the proband and his affected sister carried two heterozygote variants in *SPG7*: a common mutation [NM_003119.3:c.1529C > T (p.Ala510Val)] and novel canonical splice variant (NM_003119.3:c.1449 + 1G > A). **(D)** Linear protein domain diagram of paraplegin. The AAA+ domain is shown as two subdomains (ATPase and Lid), separated by a linker region. Three of the four variants in the SPG7 patients in this study locate to the AAA+ ATPase domain and one to the M41 peptidase domain.

Six HSP patients, three with *SPG7* mutations and three with *SPAST* mutations, were recruited to the study. These patients carried a variety of mutations (Table 1). *SPG7* patients were patient 1 [c.1454_1462del (het); c.1529C > T (het)], patient 2 [c.1529C > T (het); c.1727C > G (het)], and patient 3 [c.1449 + 1G > A (novel het); c.1529C > T (het)]. *SPAST* patients were patient 4 (c.1392A > T), patient 5 (c.583C > G), and patient 6 (c.1096G > A). All patients except patient 3 have been reported previously (Abrahamsen et al., 2013; Kumar et al., 2013). *SPG7* patient 3 was a 59-year-old man with a severe HSP phenotype complicated by upper limb cerebellar ataxia and cerebellar atrophy on MRI brain (Figures 1A,B). He was found to have the known common mutation NM_003119.3:c.1529C > T (p.Ala510Val) (Brugman et al., 2008; Bonn et al., 2010) and a novel canonical splice variant NM_003119.3:c.1449 + 1G > A, classified as pathogenic by the ACMG criteria (Richards et al., 2015) (PVS1, PM2, and PP3). Both variants were also present in a similarly affected sister (Figure 1C).

### Protein Structure

All three *SPG7* patients had a common heterozygous variant (c.1529C > T; p.A510V) in addition to different compounding heterozygous variants. All coding variants were predicted to be pathogenic by several prediction algorithms (e.g., MutationTaster, PolyPhen 2, SIFT, and PROVEAN), likely due to high evolutionary conservation of affected amino acids and the considerable physicochemical or likely structural changes imposed. Assessment of possible splicing changes for the non-coding variant using Human Splicing Finder 3.1 provided possible aberrant splicing outcomes (see below).

The pathogenicity of the c.1529C > T variant (p.A510V) common to all *SPG7* patients in this study has been extensively studied (Bonn et al., 2010). It affects a highly conserved and completely buried alanine residue at the beginning of the structural AAA+ Lid domain (Figure 1D). Because of the constrained environment around the alanine residue, the introduction of a bulkier valine side chain (89 vs. 117 Da) would be predicted to destabilize the hydrophobic core of the Lid domain. Inspecting the crystal structure of the paraplegin AAA+ ATPase domain (amino acids 305–565; PDB entry 2qz4; Karlberg et al., 2009), of the three possible conformations of the valine side chain, two are predicted to clash with Arg486, and one could potentially be accommodated. This may afford expression of the mutant p.510V protein, although it would likely be dysfunctional for ATPase activity.

The second variant in patient 1 was a deletion variant (c.1454_1462del; p.485_487delRRE) removing three charged amino acids from the highly conserved alpha helix bundle of the AAA+ Lid domain (Figure 1D). The deletion would remove an entire alpha helix turn and three charged residues necessary for structural bonds to other helices in the domain (Karlberg et al., 2009) and is predicted to cause severe structural destabilization.

The second variant in patient 2 was a missense mutation (c.1727C > G; p.S576W) located within the magnesium metal ion coordination and peptidase active site of the M41 peptidase domain (Figure 1D). The substitution of tryptophan for serine is a very large physicochemical change, a mass change of 105 to 204 Da and a hydrophilic serine residue common in active sites vs. tryptophan, which has a bulky hydrophobic aromatic side chain. This would likely cause structural and functional disruption to the peptidase active site. Based on previous mutagenesis studies of this functional site, if the variant protein were expressed, it would likely form dysfunctional complexes (Shanmughapriya et al., 2015). In this situation, peptidase activity could be compensated for by AFG3L2, as demonstrated in yeast (Bonn et al., 2010).

The second variant in patient 3 was a novel canonical donor splice site disrupting mutation (c.1449 + 1G > A) that would impact the polypeptide chain from the end of the AAA+ ATPase domain at the start of the AAA+ domain linker region (Figure 1D). A number of possibilities for the consequent splicing defect include (1) skipping of exon 10 and a frameshift leading to termination after six amino acids; (2) intronic read-through into intron 10 before an alternate donor splice site is encountered 90 nucleotides downstream, resulting in an in-frame inclusion of 30 amino acids that would alter the length of the AAA+ ATPase domain linker; and (3) intronic read-through into intron 10 before encountering a termination codon 96 nucleotides downstream. Transcript analysis would be required to resolve the resulting splicing mechanism, although the variant protein product would almost certainly not be expressed.

### Paraplegin Expression

**FIGURE 2:**
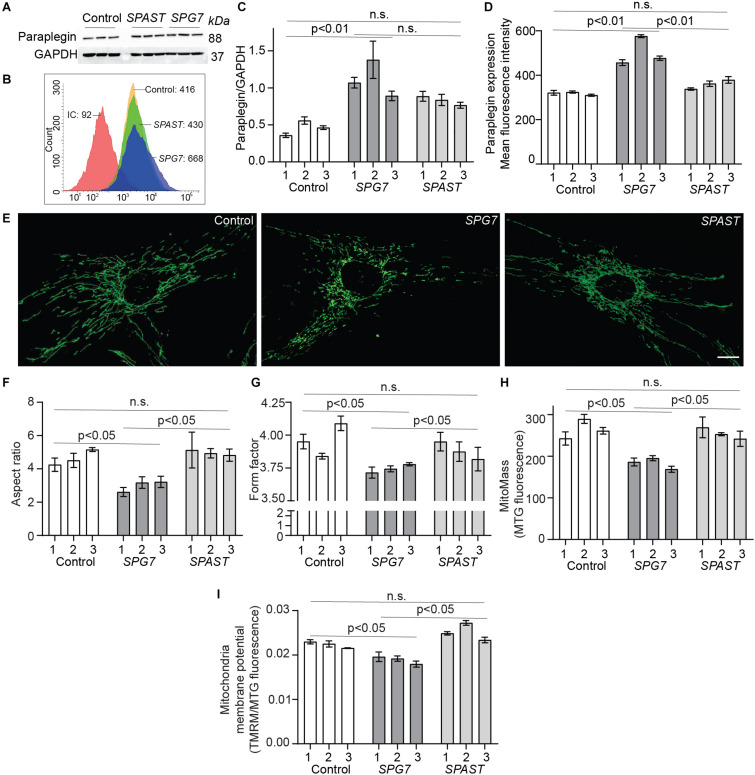
Paraplegin expression, mitochondrial morphology, and membrane potential in control, *SPG7*, and *SPAST* patient cells. **(A)** Western blot analysis of paraplegin and GAPDH expression. **(B)** Representative flow cytometry histogram of paraplegin fluorescence intensities. IC: Isotype control. **(C)** Western blot densitometry band quantification showing paraplegin expression normalized to GAPDH expression. **(D)** Paraplegin mean fluorescence intensity quantified by flow cytometry. **(E)** Representative images of MitoTracker-labeled mitochondria in cells from control, *SPG7*, and *SPAST* patient cells. Scale bar: 10 μm. **(F,G)** Mitochondria morphological analysis showing aspect ratio (mitochondria length) and form factor (degree of branching). **(H)** Mitochondrial mass was calculated as the fluorescence intensity of MitoTracker green (MTG) labeled mitochondria. **(I)** Mitochondrial membrane potential was measured using tetramethylrhodamine methyl ester (TMRM) fluorescence and normalized to mitochondrial mass (MTG fluorescence). Data presented as mean ± SEM. The bars present data from the three technical replicates. One-way analysis of variance with Tukey’s multiple comparisons test was performed to compare control, *SPG7*, and *SPAST* groups. n.s., not significant.

Paraplegin expression in patient and control cells was evaluated by Western blot analysis and by flow cytometry (Figures 2A–D). Western blot analysis showed that *SPG7* patient cells had significantly higher expression levels of paraplegin protein, compared to control cells (*p* < 0.01) (Figures 2A,C). *SPAST* patient cells had a trend of higher expression levels of paraplegin when compared to control cells, but this difference was not statistically significant (*p* = 0.06) (Figure 2C). To validate this finding, we evaluated paraplegin expression using flow cytometry. *SPG7* patient cells had significantly higher mean fluorescence intensity, compared to control cells (*p* < 0.01) and *SPAST* patient cells (*p* < 0.01) (Figures 2B,D). Both techniques showed that paraplegin expression was significantly increased in *SPG7* patient cells compared to control and *SPAST* patient cells.

### Mitochondrial Morphology

Mitochondria in *SPG7* patient cells were highly fragmented and short (low AR) with a low degree of branching and interconnectivity (low FF; Figures 2E–G), showing typical signs of a dysfunctional mitochondrial network. Conversely, mitochondria in control cells and *SPAST* patient cells were longer and interconnected (*p* < 0.05; Figures 2E–G), indicative of a healthy mitochondrial network.

Consistent with their aberrant mitochondrial morphology, *SPG7* patient cells also displayed lower mitochondrial mass (*p* < 0.05) and lower mitochondrial membrane potential (*p* < 0.05) (Figures 2H,I).

### Oxidative Phosphorylation

**FIGURE 3:**
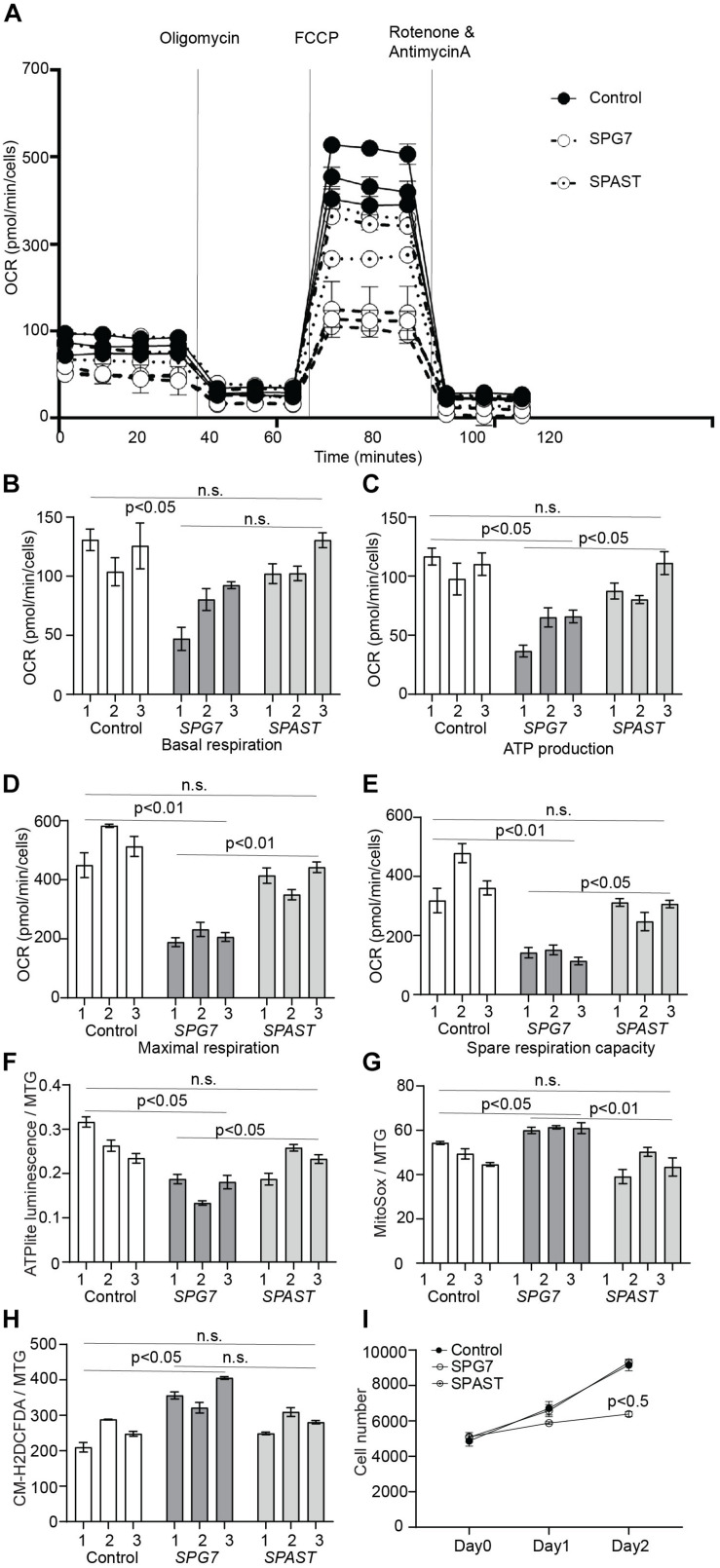
Oxidative respiration and oxidative stress in control, *SPG7*, and *SPAST* patient cells. **(A)** Representative graph of mitochondrial respiration determined by measuring OCR (mean ± SE) using the Seahorse-based assay. **(B–E)** Comparison of different aspects of mitochondrial respiration **(F)** ATP content in the cells was measured using the ATPlite assay kit and normalized to mitochondrial mass [MitoTracker green (MTG) fluorescence]. **(G)** Mitochondrial oxidative stress was measured using MitoSOX (mitochondrial superoxide indicator) fluorescence and normalized to mitochondrial mass (MTG fluorescence). **(H)** General oxidative stress was measured using the CM-H_2_DCFDA (general oxidative stress indicator) assay kit and normalized to mitochondrial mass (MTG fluorescence). **(I)** Cellular proliferation was measured for on day 0 (4 h after cell seeding), day 1, and day 2 using CyQUANT assay. Data presented as mean ± SEM. The bars present data from three technical replicates. One-way analysis of variance with Tukey’s multiple comparisons test was performed to compare control, *SPG7*, and *SPAST* groups. n.s., not significant.

To investigate if oxidative phosphorylation was impaired in HSP patient cells, we measured mitochondrial OCR using a Seahorse Bioanalyzer (Figure 3A). Compared to control and *SPAST* patient cells, *SPG7* patient cells had reduced basal respiration (Figure 3B, control vs. *SPG7*: *p* < 0.05; *SPG7* vs. *SPAST*: *p* = 0.09), significantly lower levels of oxygen consumption attributed to ATP production (Figure 3C, control vs. *SPG7*: *p* < 0.05; *SPG7* vs. *SPAST*: *p* < 0.05), reduced maximal respiration (Figure 3D, control vs. *SPG7*: *p* < 0.001; *SPG7* vs. *SPAST*: *p* < 0.01), and reduced spare respiratory capacity (Figure 3E, control vs. *SPG7*: *p* < 0.01; *SPG7* vs. *SPAST*: *p* < 0.05). Consistent with this, *SPG7* patient cells had lower total ATP content compared to both control and *SPAST* patient cells (*p* < 0.05) (Figure 3F). *SPAST* patient cells were not significantly different from control cells on any of these measures (Figures 3B–F).

### Oxidative Stress

MitoSOX fluorescence, a mitochondria-specific superoxide indicator, was significantly higher in *SPG7* patient cells compared to controls (*p* < 0.05) and *SPAST* patient cells (*p* < 0.01) (Figure 3G). CM-H_2_DCFDA fluorescence, a general reactive oxygen species indicator, was significantly higher in *SPG7* patient cells compared to control cells (*p* < 0.05) and *SPAST* patient cells (trend, *p* = 0.08) (Figure 3H).

### Cell Proliferation

MELAS and MERRF mitochondrial disease patient cells have increased oxidative stress and reduced cellular proliferation (James et al., 1996). To test if *SPG7* patient cells were similarly affected, we evaluated cellular proliferation in *SPG7*, *SPAST*, and control cells. *SPG7* patient cells had reduced cellular proliferation compared to both control and *SPAST* patient cells (*p* < 0.05) (Figure 3I).

## Discussion

We show here that *SPG7* patient ONS cells with compound heterozygous mutations have increased paraplegin expression accompanied by multiple mitochondrial dysfunctions, including aberrant mitochondrial morphology (reduced length and interconnectivity), reduced mitochondrial mass, reduced mitochondrial membrane potential, impaired oxidative phosphorylation, reduced ATP production and content, and increased oxidative stress. *SPG7* patient cells also showed reduced cellular proliferation. These cell function deficits were observed in all three *SPG7* cell lines carrying different compound heterozygous mutations including a novel *SPG7* mutation (NM_003119.3:c.1449 + 1G > A). These mitochondrial dysfunctions have previously been associated with cell and animal models lacking paraplegin (Atorino et al., 2003; Ferreirinha et al., 2004; Pareek et al., 2018). *SPAST* patient cells had a trend of lower respiratory chain function, but this was not statistically significant, indicating less severe respiratory chain dysfunction compared to *SPG7* patient cells.

In addition to spasticity, *SPG7* HSP patients generally have additional clinical features (refer to Table 1). The *SPG7* patients, but not *SPAST* patients, had clinical features typically associated with mitochondrial diseases, such as ophthalmoplegia, ptosis, ataxia, and mitochondrial cytopathy on muscle biopsy. This is in accordance with the considerable mitochondrial dysfunction in *SPG7* patient cells. These more extensive deficits in mitochondrial function may underlie the impaired cell proliferation in *SPG7* patient cells, not seen in *SPAST* patient cells.

*SPG7*, but not *SPAST*, patient cells have increased levels of mitochondrial oxidative stress and reduced oxidative phosphorylation. Consistently, dysfunctional mitochondria are often accompanied by increased production of reactive oxygen species and associated with other neurodegenerative disorders (Leadsham Jane et al., 2013; Angelova and Abramov, 2018). Paraplegin maintains mitochondrial protein quality by degrading subunits of the respiratory chain that are damaged or not assembled (Gerdes et al., 2012) and by acting as an ATP-dependent matrix-AAA protease located in the inner mitochondrial membrane that harbors the respiratory chain complexes (Quirós et al., 2015). All *SPG7* patients showed increased expression of paraplegin, despite having mutant alleles that were unlikely to be expressed. This is consistent with yeast complementation assays showing that when the AAA+ ATPase region was deleted, paraplegin was expressed but protease function was impaired (Bonn et al., 2010).

The *SPG7* patient cells in this study were compound heterozygous, of which the p.Ala510Val mutation was common to all. This variant has a carrier rate in the general population of 0.5–1% (Bonn et al., 2010; Pfeffer et al., 2014), and its association to HSP pathogenicity has been explored extensively (Karlberg et al., 2009; Bonn et al., 2010; Roxburgh et al., 2013; Pfeffer et al., 2014). In particular, an apparent increased expression of paraplegin has been observed in *SPG7* patient fibroblast cells (Pfeffer et al., 2014) and in neurites of the cerebellar and cerebral cortex on post-mortem in a patient with homozygous p.Ala510Val mutations (Thal et al., 2015). It is likely that the commonly expressed p.Ala510Val variant leads to the increased levels of paraplegin seen in our patient samples.

We have previously shown that *SPAST* patients have reduced levels of stabilized microtubules, leading to reduced organelle transport, thereby making them more sensitive to oxidative stress (Abrahamsen et al., 2013). Microtubule-dependent mitochondrial movement is necessary to maintain healthy mitochondrial structure and bioenergetics (Bartolák-Suki et al., 2017). We show here that mitochondrial function was not affected by *SPAST* mutations as severely as in *SPG7* patient cells. However, a trend toward lower respiratory chain function was observed.

We establish here that *SPG7* patient cells with increased paraplegin expression have mitochondrial dysfunction similar to patient cells that do not express paraplegin (Atorino et al., 2003). The severity of mitochondrial dysfunction in *SPG7* patient cells compared to *SPAST* patient cells suggests that the cellular mechanisms responsible for the diagnosis of HSP do not necessarily converge on mitochondrial function. Our results highlight a need for understanding the distinct biology of patient cells with different mutations. This understanding is helpful for drug screening and developing drug treatments. A common drug treatment that can rectify the reduced respiratory chain function may be useful for all *SPG7* patients.

## Data Availability Statement

All datasets presented in this study are included in the article.

## Ethics Statement

This study was reviewed and approved by the Human Research Ethics Committee of the Northern Sydney Local Health District, New South Wales Government, Australia (ethics committee reference number: RESP/15/314). The patients/participants provided written informed consent to participate in this study.

## Author Contributions

GW, AM-S, and CS conceived and designed the experiments and provided funding for the research. GW, EL, and KK performed the experiments. RD performed the protein structure and function analysis. GW performed the statistical analysis. GW, AM-S, RD, KK, and CS wrote the main manuscript text. All authors contributed to the article and approved the submitted version.

## Conflict of Interest

The authors declare that the research was conducted in the absence of any commercial or financial relationships that could be construed as a potential conflict of interest.

## Supplementary Material

FIGURE S1 The paraplegin antibody was validated using a positive control, i.e., human HEK293 cells over-expressing paraplegin protein. The paraplegin band size was 88 kDa for both the control samples tested and the positive control, showing antibody specificity.
