# Supplementary figures and images for: Mitochondrial Function in Hereditary Spastic Paraplegia: Deficits in SPG7 but Not SPAST Patient-Derived Stem Cells
Source: Front Neurosci. 2020 Aug 20;14:820. doi: 10.3389/fnins.2020.00820 (PMC7469654; doi:10.3389/fnins.2020.00820)

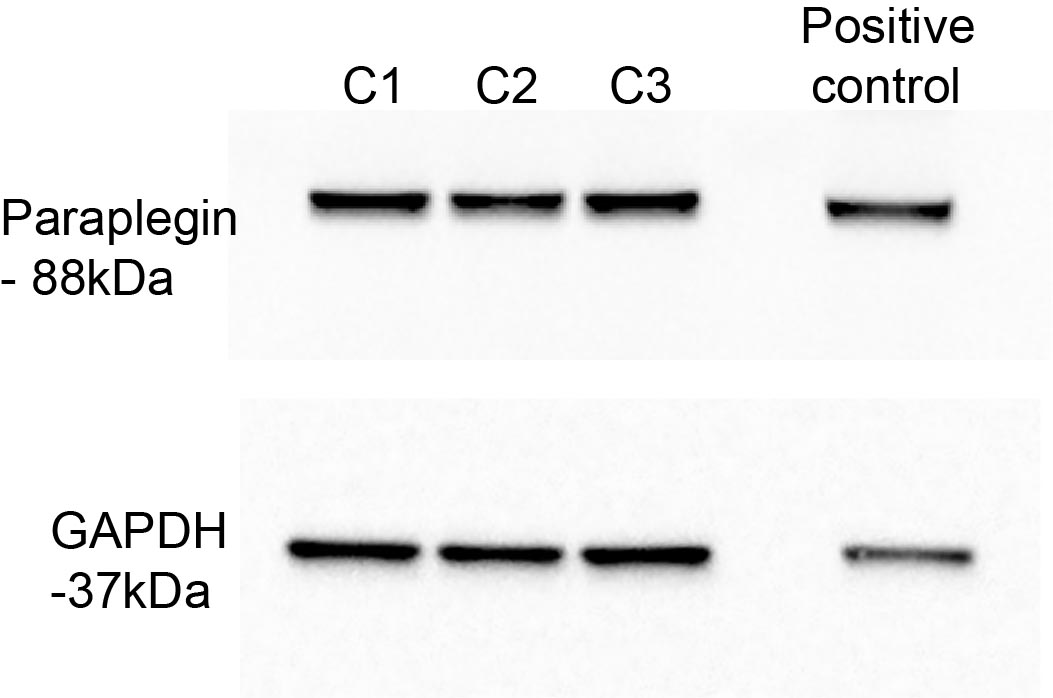

Supplement: FIGURE S1 — The paraplegin antibody was validated using a positive control, i.e., human HEK293 cells over-expressing paraplegin protein. The paraplegin band size was 88 kDa for both the control samples tested and the positive control, showing antibody specificity. [file Image_1.JPEG]
